# Using hospital registries in Australia to extend data availability on vulval cancer treatment and survival

**DOI:** 10.1186/s12885-018-4759-x

**Published:** 2018-08-30

**Authors:** David Roder, Margaret Davy, Sid Selva-Nayagam, Sellvakumaram Paramasivam, Jacqui Adams, Dorothy Keefe, Ian Olver, Caroline Miller, Elizabeth Buckley, Kate Powell, Kellie Fusco, Dianne Buranyi-Trevarton, Martin K. Oehler

**Affiliations:** 1 Centre for Population Health Research, University of South Australia, GPO Box 2471, Adelaide, SA 5001 Australia; 2 Burnside Hospital, Norwood, SA 5065 Australia; 3 Royal Adelaide Hospital Cancer Centre, Adelaide, SA 5000 Australia; 4 Flinders Medical Centre, GPO Box 2100, Adelaide, SA 5001 Australia; 5 Lyell McEwin Hospital, Haydown Road, Elizabeth Vale, SA 5112 Australia; 6 Royal Adelaide Hospital, Citi Centre Hindmarsh Square, Adelaide, SA 5000 Australia; 7 Cancer Research Institute, University of South Australia, GPO Box 2471, Adelaide, SA 5001 Australia; 8 Population Health Research Group, South Australian Health & Medical Research Institute (SAHMRI) and School of Public Health, University of Adelaide, GPO Box 11060, Adelaide, SA 5001 Australia; 9 Population Health Research Group, South Australian Health and Medical Research Institute (SAHMRI), GPO Box 11060, Adelaide, SA 5001 Australia; 10 SA Clinical Cancer Registry, SA Health, SAHMRI, North Terrace, Adelaide, SA 5000 Australia; 11 Royal Adelaide Hospital, North Terrace, Adelaide, SA 5000 Australia

**Keywords:** Vulval cancer stage treatment survival

## Abstract

**Background:**

The value of hospital registries for describing treatment and survival outcomes for vulval cancer was investigated. Hospital registry data from four major public hospitals in 1984–2016 were used because population-based data lacked required treatment and outcomes data. Unlike population registries, the hospital registries had recorded FIGO stage, grade and treatment.

**Methods:**

Unadjusted and adjusted disease-specific survival and multiple logistic regression were used. Disease-specific survivals were explored using Kaplan-Meier product-limit estimates. Hazards ratios (HRs) were obtained from proportional hazards regression for 1984–1999 and 2000–2016. Repeat analyses were undertaken using competing risk regression.

**Results:**

Five-year disease-specific survival was 70%, broadly equivalent to the five-year relative survivals reported for Australia overall (70%), the United Kingdom (70%), USA (72%), Holland (70%), and Germany (Munich) (68%). Unadjusted five-year survival tended to be lower for cancers diagnosed in 2000–2016 than 1984–1999, consistent with survival trends reported for the USA and Canada, but higher for 2000–2016 than 1984–1999 after adjusting for stage and other covariates, although differences were small and did not approach statistical significance (*p* ≥ 0.40). Surgery was provided as part of the primary course of treatment for 94% of patients and radiotherapy for 26%, whereas chemotherapy was provided for only 6%. Less extensive surgical procedures applied in 2000–2016 than 1984–1999 and the use of chemotherapy increased over these periods. Surgery was more common for early FIGO stages, and radiotherapy for later stages with a peak for stage III. Differences in treatment by surgery and radiotherapy were not found by geographic measures of remoteness and socioeconomic status in adjusted analyses, suggesting equity in service delivery.

**Conclusions:**

The data illustrate the complementary value of hospital-registry data to population-registry data for informing local providers and health administrations of trends in management and outcomes, in this instance for a comparatively rare cancer that is under-represented in trials and under-reported in national statistics. Hospital registries can fill an evidence gap when clinical data are lacking in population-based registries.

## Background

Cancers of the vulva are comparatively rare and receive less attention in Australian statistical reports than other gynaecological cancers [1, 2]. Numbers of vulval cancers diagnosed annually in Australia approximated 264 in 2003–08, with about nine in 10 of them being squamous cell carcinomas (83%) and adenocarcinomas (9%), and half affecting women aged 70 years or more [1, 3]. Five-year relative survival in Australia is 70%, which is equivalent to corresponding survival estimates of between 68 and 72% for England, the USA, Holland [3–6], and Germany (Munich) [7, 8].

Predictably, survival from vulval cancer is strongly related to stage at diagnosis, with USA Surveillance, Epidemiology, and End Results (SEER) data showing a five-year relative survival of 86% for local spread, 57% for regional spread, and 17% when distant metastases are found at diagnosis [9]. Monitoring treatment of vulval cancers and survival outcomes by stage at diagnosis has been restricted in Australia by a lack of population-based data on stage and treatment in national datasets [10]. This deficiency limits comparisons of treatment with recommended practice. Corresponding data gaps are now being addressed at a population level in Australia for leading cancer sites, but not for vulval cancers [10]. Treatment and risk-adjusted survival data are also lacking in most local clinical settings, which reduces opportunities for local evaluation [10].

Recent reports from the United States and Canada indicate secular reductions in survival from squamous cell carcinomas of the vulva across a broad age range [4]. The reasons are not known although reference was made in these countries and Germany (Munich Registry) to a change in therapy towards multimodal therapies and more conservative surgery [8, 9]. By comparison, stable survival has been reported for Holland and increases for England and Norway [6, 11, 12]. Investigating reasons for these differences requires data of greater clinical detail than routinely available in population-based cancer registries.

This report presents hospital-registry data from four major hospitals in South Australia (one of eight Australian states/territories) [13]. The data are collected to provide local services and administrations with a health-system perspective of treatment and survival by patient and tumour descriptors. The data include stage and treatment, which are complementary to population-based registry data in Australia [13].

A previous benchmark study at these hospitals in 1984–1998 indicated that vulval cancers were mostly treated by surgery, with less than a quarter having radiotherapy and only about one in 50 having systemic therapy [13]. Our hypothesis, based on treatment guidelines [14], is that a trend towards less extensive surgery and increased use of adjuvant therapies has occurred. While the international evidence on survival trends is mixed, [4, 6–8, 11, 12] the potential for a decrease in survival and a trend towards less extensive surgery will be explored, as reported for Canada and the USA [4, 7, 8].

Another aim is to assess the value of hospital-registry data for summarising trends over time in local health-service treatment and outcomes for a comparatively rare cancer. The study included 383 invasive cancers of the vulva diagnosed at our four study hospitals in 1984–2016. Although not population-based, data for these hospitals are of direct interest to local hospital services and health administrations [10, 13].

## Methods

Operations of the South Australian Clinical Cancer Registry (SACCR) have been described in SA Cancer Registry reports [13]. Research ethics approval for this study was provided by the South Australian Human Research Ethics Committee. The SACCR is authorised under Section 64, Part 7 of the South Australian Health Care Act (2008) to support the quality assurance of cancer services [15].

Postcode of residence was registered to indicate: (1) socio-economic quartile, using the Socio-Economic Indexes for Areas (SEIFA) Index of Relative Socioeconomic Disadvantage; and (2) geographic remoteness (classified as metropolitan, regional and remote) [16, 17]. These variables were chosen to investigate and adjust for potential confounding from socio-demographic associations with treatment and survival.

Person characteristics analysed included: age at diagnosis (broadly categorized due to small numbers as < 50, 50–69, 70–79 and 80+ years); SEIFA index of relative socioeconomic disadvantage (4 ordinal categories); and geographic remoteness (3 ordinal categories). Tumour characteristics (i.e., histology type, International Federation of Gynecology and Obstetrics (FIGO) stage and differentiation) were classified as shown in Table 1 [13]. FIGO staging criteria recommended in 2009 were used throughout to achieve a consistent series [18]. Staging was limited to the four major stage categories due to small numbers.

Primary site was coded using the C51 code of the International Classification of Diseases for Oncology (ICD-O Version 3). More detailed coding by vulval sub-site was not available. Histology types were extracted using ICD-O-3 morphology codes. They comprised squamous cell carcinomas (all but 11%) and adenocarcinomas, basal cell lesions, and other and unspecified histology types, which were broadly classified as squamous cell carcinomas and “other” for this study due to small numbers. Melanomas and sarcomas were excluded.

First-round treatment was defined in this study as the range of initial treatment following diagnosis. It generally took place within a 6–12-month period and was classified according to treatment by surgery (excluding procedures for diagnostic purposes only), radiotherapy and chemotherapy. Surgery type was classified using the USA Facility Oncology Registry Data Standards Facility Oncology Registry Data Standards (FORDS) classification as local excision (codes 22 & 27), hemi-vulvectomy (code 30), vulvectomy (+/− removal of inguinal lymph nodes) (code 40), or more radical excision (code 60).

Death data were extracted from the South Australian population-based cancer registry which used official death files, and for deaths occurring outside of South Australia, the National Death Index at the Australian Institute of Health and Welfare, as data sources [13]. Underlying causes-of-death were corrected when clinical data available to the registry indicated this to be appropriate [13]. The extent of loss to follow-up of deaths has been checked on many occasions through active tracing and comparison with external case series, and found to be minimal, with little effect on calculated survivals [13, 17, 19].

Disease-specific survival was calculated using Kaplan-Meier product-limit estimates, with a censoring of live patients on December 31st, 2016 [20, 21]. This method was preferred to relative survival because risks of deaths from competing causes could not be assumed to be equivalent to population norms (an underlying assumption for relative survival) due to the referral of high-risk patients (including those with extensive co-morbidity) to the referral centres covered by the SACCR [13].

Population-based data have shown disease-specific survival, based on South Australian registry coding, to be a good proxy for relative survival in many studies [13, 17]. This local validation is important because cause-specific survival can be vulnerable to variations in cause-of-death coding [22].

Cox proportional hazards regression was used to analyse differences in disease-specific survival by socio-demographic and clinical characteristics in a multivariable context, using the same follow-up period and censoring rules as for the Kaplan-Meier analyses [20, 21]. Assumptions underlying Cox regression analyses, including proportionality and lack of co-linearity, were tested and found to be met. When competing risk regression was substituted for disease-specific Cox proportional hazards regression, the results were similar [data not shown] [20, 21].

First-round treatment was analysed by person and tumour characteristic using the Pearson chi-square statistic, Mann-Whitney U Test or Spearman rank correlation, depending on whether variables were distributed on binary, nominal or ordinal scales [20, 21]. Multiple logistic regression analyses were also used to check for confounding, effect modification and clustering by treatment centre [20, 21], but did not show statistically significant effects [20].

## Results

### Descriptive

The study included 383 vulval cancers, 228 (60%) of them diagnosed in patients aged 70 years or more. Most were squamous cell carcinomas (89.0%), with the others comprising adenocarcinomas, basal cell lesions or cancers of unspecified histology type.

FIGO stage was recorded for 96%, comprising: stage I - 46%; stage II - 28%; stage III - 19%; and stage IV - 8%. Stage varied by age at diagnosis (*p* = 0.005), with the percentage classified as stage III or IV increasing from 15% for < 50 years to 26% for 50–69 years and 30% for 70+ years. There was also a difference in stage by diagnostic period with the percentage classified as stage III or IV increasing from 20% in 1984–1999 to 33% in 2000–2016 (*p* = 0.005).

### Survival

#### Unadjusted

Disease-specific survival was 70% at five years and 62% at 10 years from diagnosis in 1984–2016 (Table 1). There was a marked reduction in survival with: (1) increasing stage (*p* < 0.001), with five-year survival reducing from 86% for stage I to 28% for stage IV; (2) higher tumour grade (*p* < 0.001), with five-year survival ranging from 80% for well differentiated to 47% for poorly and undifferentiated lesions; and (3) older age at diagnosis (*p* < 0.001), with the five-year survival ranging from 91% for < 50 years to 59% for 70–79 years and 64% for 80+ years. Survival did not vary between squamous cell carcinomas and other histology types combined (*p* = 0.955), the five-year figure being 70% and 71% respectively. While five-year survival tended to be lower for cases diagnosed in 2000–2016 than 1984–1999 at 68% and 72% respectively, the difference did not approach statistical significance (*p* = 0.498). No difference in survival was observed by residential area, classified by geographic remoteness (*p* = 0.534) or relative socioeconomic disadvantage (*p* = 0.684).

#### Adjusted

Multiple logistic regression analysis confirmed a lower survival with: (1) higher stage - hazards ratio (95% confidence limits) increasing to 7.29 (3.94, 13.49) for stage IV compared with stage I; (2) higher grade - hazards ratio increasing to 1.71 (1.06, 2.76) for poorly or undifferentiated lesions compared with the well differentiated; and (3) older age at diagnosis - hazards ratio increasing to 3.14 (1.65, 6.00) for 70–79 years and 2.25 (1.16, 4.40) for 80+ years compared with < 50 years) (Table 1). A lower risk of death applied to patients diagnosed in 2000–2016 than 1984–1999 after adjusting for the other variables shown in Table 1, but the reduction in hazards ratio to 0.88 (0.61, 1.27) was not statistically significant (*p* = 0.239). Neither the geographic measure of remoteness nor socioeconomic status showed consistent survival gradients, and confidence intervals overlapped (Table 1). The elevated hazards ratio for highly remote areas of 1.40 (0.76, 2.59) was based on only 24 cases.

### Any treatment

#### Unadjusted

Overall, 98% of patients were recorded to have received some treatment for their cancer (i.e., surgery, radiotherapy and/or chemotherapy). The distribution by treatment combination was: surgery only – 72%; surgery and radiotherapy – 16%; radiotherapy only – 4%; surgery, radiotherapy and chemotherapy – 5%; and other (radiotherapy & chemotherapy or chemotherapy alone) – 1%; and no treatment – 2%. Treatment patterns varied between 1984 and 1999 and 2000–2016 (*p* = 0.001), with a reduced proportion in 2000–2016 having surgery only and a higher proportion having combination surgery, radiotherapy and chemotherapy or, less so, radiotherapy and chemotherapy.

The proportion having any treatment did not vary by: age (*p* = 0.140), ranging from 99% for ages < 70 years to 96% for patients aged 80+ years; grade (*p* = 0.368); histology type (*p* = 0.560); diagnostic period (*p* = 0.124); or residential area classified by geographic remoteness (*p*= 0.417). Borderline differences applied to: stage (*p*= 0.052), with the proportion having any treatment decreasing from 99% for stages I and II to 96% for stages III and IV; and socioeconomic status (*p* = 0.061), with the proportion having treatment being 100% for low and 97% for each other category.

#### Adjusted

Adjusted analysis, including all variables in Table 1 as predictors of treatment, did not show statistically significant differences in proportions receiving any treatment by: age; stage; grade; histology type; geographic measures of socioeconomic status or remoteness, or diagnostic period (*p* > 0.200) (note: although a downward trend in odds of treatment (OR) was suggested for higher stage (OR 0.94 for stage II, 0.19 for stage III, and 0.39 for stage IV compared with the stage I reference category).

**Table 1:** % case survival from cancer of the vulva by period post-diagnosis; South Australian major public hospitals, 1984–2016 diagnoses^a^

Number of cases	Surv 1 yr	Surv 2 yr	Surv 5 yr	Surv 10 yr	Surv 20 yr	*p* value**	Hazard ratio^b^ (95% CLs) -adjusted
All (*n* = 383)	84.1	75.7	70.0	61.5	55.3		
Age at diagnosis (yrs.):							
< 50 (*n* = 56)	96.4	91.1	91.1	82.8	75.6	*p* < 0.001	1.00
50–69 (*n* = 100)	86.6	83.5	77.4	65.6	57.6		2.10 (1.06, 4.17)
70–79 (*n* = 113)	82.0	66.6	59.1	51.9	45.0		3.14 (1.65, 6.00)
80+ (*n* = 114)	77.8	70.5	63.6	56.1	52.6		2.25 (1.16, 4.40)
FIGO stage:							
I (*n* = 167)	94.5	92.0	85.8	77.7	70.1	*p* < 0.001	1.00
II (*n* = 103)	92.2	81.5	71.6	61.4	55.5		1.62 (1.04, 2.53)
III (*n* = 68)	68.3	43.5	41.8	34.1	28.9		3.89 (2.47, 6.13)
IV (*n* = 29)	27.6	27.6	27.6	27.6	–		7.29 (3.94, 13.49)
(Unknown (*n* = 16))	(93.8)	(93.8)	(93.8)	(76.7)	(68.2)		(1.06 (0.37, 3.09))
Differentiation:							
Well (*n* = 102)	93.1	86.1	79.9	69.9	60.3	*p* < 0.001	1.00
Moderate (*n* = 136)	84.1	74.8	70.1	62.5	56.2		0.80 (0.51, 1.25)
Poorly/undifferentiated (*n* = 75)	69.1	54.0	46.9	41.9	40.1		1.71 (1.06, 2.76)
(Unknown (*n* = 70)	(86.8)	(85.3)	(79.8)	(66.8)	(63.6)		(0.94 (0.51, 1.71))
Histology:							
Squamous cell carcinoma (*n* = 341)	85.1	76.0	69.8	61.1	55.1	*p* = 0.955	1.00
Other (*n* = 42)	75.9	73.4	70.9	64.1	57.4		0.69 (0.40, 1.21)
Socioeconomic (SEIFA):							
Low (*n* = 138)	84.5	76.8	74.3	66.7	59.2	*p* = 0.684	1.00
Low/med (n = 75)	87.7	80.8	73.7	55.5	55.5		1.25 (0.78, 2.02)
Med/high (*n* = 77)	88.1	77.4	68.7	58.7	53.2		1.19 (0.76, 1.88)
High (*n* = 93)	77.4	68.7	61.8	61.8	52.0		1.14 (0.73, 1.76)
Geographic remoteness:							
Low (*n* = 314)	83.5	75.0	69.1	60.8	55.0	*p* = 0.534	1.00
Moderate (*n* = 45)	86.0	76.5	76.5	70.4	62.8		1.00 (0.56, 1.79)
High (*n* = 24)	87.5	83.3	70.1	56.1	48.1		1.40 (0.76, 2.59)
Diagnostic period (calendar years.):							
1984–99 (*n* = 199)	83.9	76.4	71.9	63.3	56.7	*p* = 0.498	1.00
2000–16 (*n* = 184)	84.6	74.9	67.6	59.3	–		0.88 (0.61, 1.27)

**Derived from unadjusted Cox proportional hazards regression

^a^Kaplan-Meier product-limit disease-specific estimates; date of censoring of live cases - Dec 31, 2016

^b^Derived from Cox proportional hazards regression, adjusting for other variables in the Table

### Surgery

#### Unadjusted

Approximately 94% of patients had some type of surgical treatment (Table 2). The proportion decreased with: (1) increasing stage (*p* < 0.001) from 98% for stage I to 79% for stage IV; (2) higher tumour grade (*p* = 0.002), decreasing from 99% for well differentiated to 91% for the poorly and undifferentiated lesions; and (3) higher socioeconomic status (*p* = 0.021), decreasing from 96% for low to 88% for high. Age at diagnosis, histology type, geographic remoteness, and diagnostic period were not associated with the proportion having surgery (*p* ≥ 0.165).

Surgery type was recorded for 90% of surgical cases, indicating that 25% had a local excision, 31% a hemi-vulvectomy, 17% a total vulvectomy, and 27% a more radical excision. There was a difference by diagnostic period (*p* < 0.001), with the proportion having total vulvectomy or a more radical procedure reducing from 49% for 1984–1999 to 37% for 2000–2016. Differences in surgery type were not found by age (*p* = 0.806) or stage (*p* = 0.225).

#### Adjusted

Multivariable analysis confirmed the lower odds ratios for having surgery of some type with: (1) increasing stage - OR reducing to 0.11 (0.02, 0.53) for stage IV compared with stage I; and (2) higher grade - OR reducing to 0.22 (0.02, 0.53) for poorly and undifferentiated compared with well differentiated lesions (Table 2). Differences were not indicated by age, histology type, diagnostic period or residential area classified by remoteness (*p* ≥ 0.150). The adjusted analysis confirmed that the odds of surgery were lower for high than low socioeconomic areas, but the odds ratio was 0.33 (0.10–1.13) which was not statistically significant (*p* > 0.05).

**Table 2:** % cancers of the vulva treated by surgery (and odds ratios for surgery) as part of the primary course of treatment; Australian major public hospitals, 1984–2016 diagnoses

Number of cases	Surgery (%)	*p* value* Mann-Whitney or Fisher Exact Test	Odds ratios^a^ - adjusted (95% CIs)
All (*n* = 383)	93.7	–	–
Age at diagnosis (yrs.):			
< 50 (*n* = 56)	98.2	MW *p* = 0.452	1.00
50–69 (*n* = 100)	91.0		0.28 (0.03, 2.58)
70–79 (*n* = 113)	95.6		0.64 (0.06, 6.38)
80+ (*n* = 114)	92.1		0.30 (0.03, 2.77)
FIGO stage:			
I (*n* = 167)	98.2	MW *p* = < 0.001 (excluding UK)	1.00
II (*n* = 103)	97.1		0.70 (0.13, 3.87)
III (*n* = 68)	83.8		0.13 (0.03, 0.53)
IV (*n* = 29)	79.3		0.11 (0.02, 0.53)
(Unknown (*n* = 16))	(93.8)		(0.76 (0.07, 8.87))
Differentiation:			
Well (*n* = 102)	99.0	MW *p* = 0.002 (excluding UK)	1.00
Moderate (*n* = 136)	94.1		0.25 (0.03, 2.28)
Poorly/undifferentiated (*n* = 75)	90.7		0.22 (0.02, 0.53)
(Unknown (*n* = 70)	(88.6)		(0.13 (0.01, 1.28))
Histology:			
Squamous cell carcinoma (*n* = 341)	94.4	FET *p* = 0.165	1.00
Other (*n* = 42)	88.1		0.59 (0.16, 2.13)
Socioeconomic (SEIFA):			
Low (*n* = 138)	96.4	MW *p* = 0.021	1.00
Low/med (*n* = 75)	94.7		0.58 (0.13, 2.64)
Med/high (*n* = 77)	94.8		0.69 (0.15, 3.05)
High (*n* = 93)	88.2		0.33 (0.10, 1.13)
Geographic remoteness:			
Low (*n* = 314)	93.6	MW *p* = 0.757	1.00
Moderate (*n* = 45)	91.1		0.35 (0.09, 1.40)
High (*n* = 24)	100.0		0.00 (0.00, 3.26)
Diagnostic period (calendar years.):			
1984–99 (*n* = 199)	95.5	FET *p* = 0.205	1.00
2000–16 (*n* = 184)	91.8		0.79 (0.28, 2.20)

*Derived from Mann-Whitney U Test (ordinal) or Fisher Exact Test (binary)

^a^Derived from multiple logistic regression, adjusting for other variables in the Table

### Radiotherapy

#### Unadjusted

Radiotherapy was used in the treatment of 26% of these cancers. The proportion having radiotherapy increased with: more advanced stage (*p* < 0.001), from 7% for stage I to 64% for stages III and IV; higher grade (*p* = 0.001), with the proportion ranging from 12% for well differentiated to 43% for poorly and undifferentiated lesions; and diagnostic period (*p* = 0.015), with a proportion of 21% for 1984–99 and 32% for 2000–16 (Table 3). Differences were not observed by age at diagnosis, histology type or residential area classified by socioeconomic status or remoteness (*p* ≥ 0.098).

#### Adjusted

Multivariable analysis indicated lower odds ratios of radiotherapy with increasing age (OR decreasing to 0.18 (0.06, 0.48) for 80+ years compared with < 40 years) (Table 3). Increasing odds were indicated for: more advanced stage, with an OR of 32.11 (13.24, 77.85) for stage III and 25.37 (8.54, 75.37) for stage IV compared with stage I; and higher grade, with an OR of 2.67 (1.06, 6.68) for poorly and undifferentiated lesions compared with the well differentiated. Differences were not observed by histology type, diagnostic period, or geographic measures of remoteness or socioeconomic disadvantage (*p* ≥ 0.278) (Table 3).

**Table 3:** % cancers of the vulva having radiotherapy (and odds ratios for radiotherapy) as part of the primary course of treatment; Australian major public hospitals, 1984–2016 diagnoses

Number of cases	Radiotherapy (%)	*p* value* Mann-Whitney or Pearson Chi-square	Odds ratios^a^ - adjusted (95% CIs)
All (*n* = 383)	26.4	–	–
Age at diagnosis (yrs.):			
< 50 (*n* = 56)	30.4	MW *p* = 0.098	1.00
50–69 (*n* = 100)	25.0		0.41 (0.16, 1.06)
70–79 (*n* = 113)	35.4		0.70 (0.28, 1.75)
80+ (*n* = 114)	16.7		0.18 (0.06, 0.48)
FIGO stage:			
I (*n* = 167)	7.2	MW *p* < 0.001 (excluding UK)	1.00
II (*n* = 103)	24.3		5.97 (2.60, 13.72)
III (*n* = 68)	66.2		32.11 (13.24, 77.85)
IV (*n* = 29)	58.6		25.37 (8.54, 75.37)
(Unknown (*n* = 16))	(12.5)		(1.81 (0.33, 10.05))
Differentiation:			
Well (*n* = 102)	11.8	MW *p* = 0.001 (excluding UK)	1.00
Moderate (*n* = 136)	28.7		1.87 (0.81, 4.33)
Poorly/undifferentiated (*n* = 75)	42.7		2.67 (1.06, 6.68)
(Unknown) (*n* = 70)	(25.7)		(2.18 (0.80, 5.98))
Histology:			
Squamous cell carcinoma (*n* = 341)	26.4	*p* = 0.978	1.00
Other (*n* = 42)	26.2		0.87 (0.32, 2.36)
Socioeconomic (SEIFA):			
Low (*n* = 138)	26.8	MW *p* = 0.565	1.00
Low/med (*n* = 75)	17.3		0.63 (0.25, 1.56)
Med/high (*n* = 77)	32.5		1.60 (0.74, 3.45)
High (*n* = 93)	28.0		0.77 (0.36, 1.64)
Geographic remoteness:			
Low (*n* = 314)	25.5	MW *p* = 0.380	1.00
Moderate (*n* = 45)	28.9		2.29 (0.53, 3.13)
High (*n* = 24)	33.3		1.38 (0.44, 4.36)
Diagnostic period (calendar years.):			
1984–99 (*n* = 199)	21.1	*p* = 0.015	1.00
2000–16 (*n* = 184)	32.1		1.72 (0.93, 3.15)

*Derived from Mann-Whitney (ordinal) or Pearson chi-square (1d.f.) (binary)

^a^Derived from multiple logistic regression, adjusting for other variables in the Table

### Chemotherapy

#### Unadjusted

Only 6% of patients received systemic therapy, with this percentage reducing with increasing age (*p* < 0.001) and increasing with more advanced stage (*p* = 0.002) (Table 4). A greater use of chemotherapy applied in 2000–2016 than 1984–1999 (*p* < 0.001). Differences were not indicated by grade, histology, or geographic area classified by socioeconomic status or remoteness (*p* ≥ 0.080). During 1984–1999, chemotherapy protocols where not generally recorded. In 2000–2016, protocols were reported for 85%, all of them involving cisplatin which was provided together with radiotherapy.

#### Adjusted

Adjusted analyses confirmed: the lower odds of chemotherapy for patients aged 70–79 and 80+ years compared with < 40 years as the reference; and the higher odds of chemotherapy for stages III and IV than the stage I reference, and for the 2000–2016 than 1984–1999 (Table 4). Higher odds of chemotherapy were also indicated for highly remote than metropolitan residential areas (OR 10.72 (1.32, 87.37)) and potentially, for high compared with low socioeconomic areas (OR 4.97 (1.00, 24.87). Differences were not indicated by histology type or grade (Table 4).

**Table 4:** % cancers of the vulva having chemotherapy (and odds ratios for chemotherapy) as part of the primary course of treatment; Australian major public hospitals, 1984–2016 diagnoses

Number of cases	Systemic therapy (%)	*p* value* Mann-Whitney or Fisher Exact Test	Odds ratios^a^ adjusted (95% CIs)
All (*n* = 383)	6.0	–	–
Age at diagnosis (yrs.):			
< 50 (*n* = 56)	14.3	MW *p* < 0.001	1.00
50–69 (*n* = 100)	8.0		0.36 (0.08, 1.56)
70–79 (*n* = 113)	6.2		0.21 (0.05, 0.95)
80+ (*n* = 114)	0.0		0.00 (0.00, 0.30)
FIGO stage:			
I (*n* = 167)	3.0	MW *p* = 0.002 (excluding UK)	1.00
II (*n* = 103)	1.9		1.46 (0.22, 2.53)
III (*n* = 68)	17.6		18.77 (3.83, 92.02)
IV (*n* = 29)	13.8		9.98 (1.58, 63.24)
(Unknown (*n* = 16))	(0.0)		(0.00 (0.00, 5.78))
Differentiation:			
Well (*n* = 102)	3.9	MW *p* = 0.080 (excluding UK)	1.00
Moderate (*n* = 136)	5.1		0.47 (0.09, 2.53)
Poorly/undifferentiated (*n* = 75)	10.7		1.21 (0.22, 6.60)
(Unknown) (*n* = 70)	(5.7)		(0.67 (0.12, 3.75))
Histology:			
Squamous cell carcinoma (*n* = 341)	6.5	FET *p* = 0.492	1.00
Other (*n* = 42)	2.4		0.31 (0.03, 3.48)
Socioeconomic (SEIFA):			
Low (*n* = 138)	8.7	MW *p* = 0.795	1.00
Low/med (*n* = 75)	0.0		0.00 (0.00, 0.75)
Med/high (*n* = 77)	3.9		0.74 (0.12, 4.71)
High (*n* = 93)	8.6		4.97 (1.00, 24.87)
Geographic remoteness:			
Low (*n* = 314)	5.4	MW *p* = 0.223	1.00
Moderate (*n* = 45)	4.4		0.26 (0.02, 2.87)
High (*n* = 24)	16.7		10.72 (1.32, 87.37)
Diagnostic period (calendar years.):			
1984–99 (*n* = 199)	1.5	FET *p* < 0.001	1.00
2000–16 (*n* = 184)	10.9		8.54 (2.14, 34.02)

*Derived from Mann-Whitney U Test (ordinal) or Fisher Exact Test (binary)

^a^Derived from multiple logistic regression, adjusting for other variables in the Table

## Discussion

The five-year survival of 70% for vulval cancer in this study was the same as the 70% relative survival estimate for Australia overall for 1982–2010 and broadly equivalent to the corresponding 71% for the USA (SEER data, 2009–13), 70% for Holland, 70% for the United Kingdom, and 68% reported by the Munich Cancer Registry [3, 4, 6–8, 11].

Survival by stage was also similar to international comparators, as shown by SEER staging. Compared with the USA (2007–13), in our study five-year survival was: for local disease (FIGO stage I), the same at 86% for the USA; and for regional spread (FIGO stages II & III), similar at 60% compared with 57%; but for distant disease (FIGO stage IV), higher at 28% Vs 17% in the USA [9]. The better result for stage IV in this study is reassuring, but interpretation should be cautious due to the potential for artificial effects. While gains in chemotherapy or other treatments may have contributed, the potential for measurement effects from stage shift due to the use of more sensitive diagnostic technologies or from variations in registry methodology cannot be discounted.

Survival was relatively stable across 1984–2016. While the unadjusted five-year estimate for 2000–2016 was marginally lower than for 1984–1999, the difference did not approach statistical significance in the unadjusted comparison; moreover, it was not confirmed in the multivariate analysis where a contrary non-significant increase in survival was observed. Overall, little change was apparent, as reported for Dutch women [6]. In contrast, survival gains have been reported for England and Norway [11, 12], and decreases for the USA and Canada [4].

Notably the percentage of patients with stage III or IV disease was higher at 33% in 2000–2016 compared with 20% for 1984–1999 (*p* = 0.005). This may have been affected by changes in patient referral practices, or increased detection of distant metastases through advances in imaging. Whatever the cause, with a third of lesions diagnosed at an advanced stage, the potential to increase survival through earlier detection should be considered.

The lack of variation in survival by geographic remoteness of residential area and socioeconomic status suggests equity in service delivery. In general, differences in treatment were not found by location of residence, although a greater use of chemotherapy was suggested for patients living in geographically remote areas and potentially for upper socioeconomic areas. Where provided, chemotherapy generally accompanied radiotherapy, consistent with its role as a radiotherapy sensitizer [23]. While access to radiotherapy centres in metropolitan areas would be more difficult for many residents of remote areas, patients of the present study hospitals appeared to have an equivalent uptake of radiotherapy, irrespective of place of residence.

A trend towards more conservative surgery was evident, as reported for Canada, the USA, Germany (Munich) and Holland [4, 6–8]. This may contribute to reduced treatment morbidity and help maintain continence and sexual function [24]. It is reassuring that more conservative surgery has also taken place in the present study hospitals without evidence of a downward trend in survival.

An increased incidence of vulval cancer has been reported for many countries, including Australia, especially in younger women where human papillomavirus (HPV) infection (often with a history of a prior HPV-related lesion) is thought to play a stronger role [3, 25]. This is in contrast with older women where risk factors may include smoking, immunosuppression or a history of lichen sclerosis [24]. The present data are consistent with a larger incidence increase in younger women [3, 25], insofar as a younger age distribution of vulval cases applied for the 2000–2016 than 1984–1999 diagnostic period (*p* = 0.022) (the percentage < 70 years being 48% and 34% respectively) [3, 25]. The effects of any changes in the aetiology of these cancers on survival could not be addressed with the data available for this study.

Our results show a lower survival from vulva cancer in older age, but this is seen for many cancer sites [1, 2]. We have no evidence to suggest whether it was influenced by age-related differences in cancer biology. It is likely that increased frailty and comorbidity would be involved [26], which may have contributed to the lower uptake of adjuvant therapies in older than younger patients. Surgical excision also may be more difficult in older cases when lesions are more broadly spread.

This study illustrates the use of hospital-registry data for profiling trends in clinical management and survival for local hospital settings. Although lacking information on recurrence, these data are of great interest to clinicians and local health service administrations, and they complement population-registry data with information on stage and treatment. Further, the study illustrates the value of hospital registries for tracking practices and outcomes across decades, including in this instance for a comparatively rare cancer that is under-represented in clinical trials and under-reported in national cancer statistics [1, 2].

## Conclusions

1. Survival from vulval cancers treated at these hospitals is equivalent to survival outcomes observed Australia-wide and in the USA (SEER data), Holland, England, Norway and Germany (Munich).
2. The proportion recorded as diagnosed at a more advanced stage has increased, which may reflect increased detection of more advanced disease, including metastases. With about a third now being diagnosed at an advanced stage, there may be opportunities to improve survival through earlier diagnosis, and the detection and treatment of precursor lesions.
3. Survival appears to have been stable across 1984–2016. Stable survival has also been reported for Holland and Germany (Munich), contrary to the increases reported for Norway and England, and decreases for the USA and Canada.
4. A trend towards more conservative surgical management is evident, with a greater use of adjuvant therapy for more advanced disease. The use of more conservative surgery may have reduced side effects and enhanced quality of life, although data were not available in this study to investigate those aspects.
5. Equity in service delivery is apparent. While chemotherapy appeared to be more common for women from very remote areas, there were in general few observed differences in treatment and survival by geographic measures of remoteness and socioeconomic status.
6. Older women generally receive less surgical intervention and have a lower survival. This may reflect difficulties in excising lesions that are more widespread. Also compromising treatment plans to accommodate reducing patient resilience with increasing age involves complex decision-making and uncertainty where additional research and protocol development are needed.
7. This study demonstrates the value of clinical registries in complementing population-based registries for evaluating service activity and survival outcomes in local service settings. The data for vulval cancer are particularly welcomed, since this is a comparatively rare cancer that is under-represented in clinical trials and under-reported in national cancer statistics.

## Abbreviations

FIGO: International Federation of Gynecology and Obstetrics
FORDS: Facility Oncology Registry Data Standards
HPV: Human papillomavirus
ICD-O Version 3: International Classification of Diseases for Oncology
SACCR: South Australian Clinical Cancer Registry
SEER: Surveillance, Epidemiology, and End Results
SEIFA: Socio-Economic Indexes for Areas
SNOMED: Systematized Nomenclature of Medicine
